# Adaptive control for microgrid frequency stability integrating battery energy storage and photovoltaic

**DOI:** 10.1038/s41598-025-28321-x

**Published:** 2025-12-14

**Authors:** Hossam S. Salama, Abdelfatah Ali, Karar Mahmoud

**Affiliations:** 1 Electrical Engineering Department, Faculty of Engineering, Aswan University, Aswân, 81542 Egypt; 2 Electrical Engineering Department, Faculty of Engineering, Qena University, Qena, 83521 Egypt; 3 Electrical Engineering Department, American University of Sharjah, Sharjah, United Arab Emirates

**Keywords:** Primary control implementation, Adaptive centralized secondary control, Battery energy storage system (BESS), Photovoltaic (PV) system, Microgrid (MG), Energy science and technology, Engineering

## Abstract

The integration and control of Microgrid (MG) systems remain critical challenges in the widespread adoption of renewable energy sources, especially photovoltaic (PV). An adaptive control approach is proposed in this work to improve the MG stability in the presence of PV and battery energy storage systems (BESSs). The proposed approach incorporates adaptive centralized secondary control, primary control, and local PV/BESS control. The primary control based on the droop control approach is applied to regulate voltage and frequency in a decentralized manner while ensuring balanced power-sharing among different distributed generators (DGs) in the MG. Besides that, an adaptive coordinated secondary control is implemented to alleviate the deviations of frequency and voltage caused by PV intermittent generation and load variation, which has a central controller that restores nominal setpoints for all DGs. The BESS type used in this study is a lithium-ion battery which is applied to preserve the DC bus voltage approximately constant during various events, enhance system resilience against PV power intermittency, and balance load power demand. The biggest advantage of the proposed control approach is that it dynamically regulates battery charging and discharging to compensate for variations in PV generation and load demand, ensuring stable system operation. In contrast to conventional studies that assume an ideal DC source to represent DGs, this study models PV generation with real-time fluctuations and maximum power point tracking, providing a practical and realistic simulation environment. The robustness and effectiveness of the proposed technique are validated using MATLAB Software. The results obtained signify highly efficient voltage and frequency stability, improved system resilience under dynamic conditions, and optimal power-sharing among DGs. Finally, a comparative analysis with conventional models highlights the superior adaptability and reliability of the proposed approach, making it a viable solution for real-MG applications.

## Introduction

### Background

The increasing energy demand and the direction of reducing greenhouse gas emissions and transitioning to sustainable energy systems have led to significant advancements in modern power systems. Microgrids (MGs) have emerged as a promising solution to these challenges using renewable energy sources (RESs) integration, improving the resilience of power systems and energy efficiency^1^. Aligned with this context, appropriate and effective control techniques are critical for ensuring the economic viability and reliability of MGs. Nowadays, continued innovation in different control methods and system design has facilitated the widespread adoption and integration of MGs into modern power systems^2^. An MG can be defined as a localized energy system capable of operating independently or in coordination with the main electrical grid. It usually includes distributed generators (DGs), such as wind turbines, PV systems, controllable loads, and energy storage systems (ESSs). MGs provide a modular design for optimizing energy production, consumption, and distribution at the level of facility or community. The operation flexibility of MGs enables them to provide a lot of benefits^3^, such as enhancing reliability and resilience during grid disturbances or outages and supporting RES integration through real-time energy management. Moreover, it minimizes losses of transmission and distribution due to localized generation and saves economic benefits by ancillary service provision and energy cost optimization.

Regardless of the above-mentioned merits of MGs, challenges, e.g., the persistence of scalability, effective system control, and energy management system. The dynamic and decentralized nature of MGs makes necessary advanced and effective control techniques and robust coordination mechanisms to ensure optimal operation, maintain stability, and accommodate high penetration levels of RES. The increasing adoption of RESs, driven by environmental concerns and technological advancements, has further emphasized the importance of MG systems and effective control methods. The PV sources are particularly significant among various technologies of RES because of their wide range of applications. Their growing popularity is attributed to declining acquisition costs and consistent availability, making them a preferred choice for sustainable energy solutions with MG systems.

### Motivation

Based on the latest report from the International Energy Agency (IEA)^4^, RESs have experienced significant growth in the last decade. Recently, in 2022, the supply from RESs, such as PV, wind, geothermal, hydro, and ocean energy, expanded to around 8%, increasing their share in the global energy supply to 5.5%. The IEA’s “Renewables 2024” report predicts that the share of RESs belonging to the electricity sector will increase from 30% in 2023 to 46% by 2030, with solar and wind accounting for almost all this expansion. Furthermore, the IEA projects that global renewable capacity will expand by over 5,520 GW during 2024–2030, which is 2.6 times more than the deployment of the previous six years (2017–2023). The result of these statistics highlights the accelerating adoption of RESs worldwide. Wind turbines and PV systems rely on wind speed and solar irradiation as their energy sources. As a result, they are considered unreliable, leading to intermittent power generation and causing voltage and frequency instabilities in modern power systems. Controlled energy storage systems are a key solution to address the challenges associated with RESs. Their primary function in modern power systems is to balance the power exchange between RESs and the grid, enhancing network stability. To effectively mitigate issues caused by RESs and maximize their benefits, efficiency, rapid response, and longevity are crucial factors when selecting ESSs^5^. Many types of ESSs are used with MGs, such as batteries, which are considered the most common ESS for MGs due to scalability, flexibility, high efficiency, modularity, fast response times, and suitability for RES integration.

Batteries have a lot of types^5,6^, such as lithium-ion (Li-ion), lead-acid, sodium-sulfur, and flow batteries. However, it has limitations and challenges, such as limited lifespan, high upfront costs, and environmental concerns with disposal. Flywheels store kinetic energy by spinning a rotor, releasing it as needed with high power density, rapid response, and a long lifespan. However, they have high capital costs and limited energy storage capacity. Supercapacitors store energy electrostatically and provide short-term, high-power energy bursts. They offer a long lifecycle and fast charging/discharging but are limited by low energy density, making them suitable only for specific applications. Thermal energy storage stores excess energy as heat or cold for later use. It is ideal for heating/cooling applications and combining heat and power systems. However, it is limited to thermal applications and requires significant space. Hydrogen storage uses excess electricity to produce hydrogen via electrolysis, which can be stored and later converted back into electricity through fuel cells. It offers high energy density and long-term storage but faces challenges like high costs, low round-trip efficiency, and infrastructure requirements. Pumped Hydro Storage stores energy by pumping water to a higher elevation and releasing it to generate electricity. It provides high storage capacity and a long lifespan but requires specific geographical conditions and involves high capital investment.

### Literature review and main contributions

Microgrids have been dominated by high penetration of voltage source converter-based DERs^7–11^. As a result, several control strategies have been compared and discussed in the literature, focusing on their role in managing grid frequency^12^. Various techniques and classifications are explored in^13^, including (1) synchronous generator model-based methods, (2) swing equation-based approaches, (3) frequency-power response-based techniques, (4) droop-based control, and (5) virtual inertia-related issues. Different virtual control strategies for wind turbines are presented in^14^. A classification of voltage source converters grid-forming control techniques is conducted in^15^, while^16^ summarizes three control methodologies as grid-forming control categories. The authors of^17^ have enriched the literature review by providing an updated analysis of voltage source grid-forming control techniques, categorized into five distinct types.

On the other hand, many works have been focused on investigating the role of ESS in MGs. The authors in^18^, have discussed grid stability and reliability, emphasizing the critical role of ESSs in maintaining frequency and voltage stability. Where they have pointed out the critical role of ESSs to backup power during outages, ensuring continuous energy supply to the essential loads. Furthermore, the proposed work has explained the effective role of ESS in smoothing the output power from RESs such as PV and wind turbines, which leads to alleviating their intermittency and improving grid resilience and stability. The authors in^19^ explored peak shaving and load management, pointing out the important role of BESS based on a modified bat algorithm that is employed to optimize energy distribution and reduce grid stress, where this is achieved by enabling the BESS to charge energy during the off-peak period and discharge during the peak period. This process, making energy consumption more economical and sustainable, enhances grid efficiency and significantly reduces demand charges and operational costs. Meanwhile, reference^20^ focuses on the analysis of power quality improvement, indicating the role of BESSs in improving the grid performance incorporated with PV. It shows the successful participation of BESS to alleviate voltage and frequency fluctuations, which ensures a reliable and stable power supply and contributes to improving overall system efficiency. Additionally, the study used an optimization tool with the BESS-based differential evolution optimization (DEO) technique, showing how effective they are under different disturbance levels.

The authors in references^21,22^ discussed demand side management in terms of energy arbitrage and demand response. They focused on the strategic role of ESSs in optimizing energy costs and market participation by explaining the impact of BESS in dynamic pricing participation, where it can charge power during the low price and discharge power during the high price period. This, in turn, helps to balance supply and demand on the grid but also creates opportunities for revenue generation. Using price differentials, ESSs increase economic viability, boost return on investment, and support a more efficient and sustainable energy market.

Simulations of PV systems generally employ two distinct modeling approaches^23^. The first is the Ideal DC Source Model, where the PV source is treated as an ideal DC power supply with a constant voltage or current, disregarding variations due to irradiance, temperature, or nonlinear characteristics. The second is the Real PV Model, which incorporates actual operational behaviors, accounting for environmental factors, partial shading, and temperature effects. The selection between these models plays a crucial role in determining the accuracy, complexity, and computational efficiency of the analysis. A comparative overview of these approaches is provided in Table 1.

**Table 1:** Comparison of articles using PV as an ideal DC source vs. a real PV source in Simulation.

Feature	PV as an ideal DC source	PV as a real source
Accuracy	Simplified, lacks real-world variations	More realistic, it accounts for dynamic conditions
Computational complexity	Lower, faster simulations	Higher due to nonlinear equations
Consideration of MPPT	Often ignored or assumed optimal	Requires MPPT algorithm for efficiency
Effects of Irradiance & Temperature	Not included	Modeled dynamically based on environmental conditions
Use case	Control and stability studies	Performance evaluation, power quality analysis
Power fluctuations	Absent or minimal	Present, requiring smoothing techniques
Modeling approach	Assumed as constant voltage/current	Modeled using PV characteristic equations
Suitability	Grid stability	Renewable energy integration, real-world behavior analysis

To cover the gap in the literature, this study applies the primary control method to ensure the local stability of DERs based on the droop control technique, which is used for voltage and frequency regulation in a decentralized manner. This method balances power-sharing among distributed generators. Power sharing among inverters in a MG system is crucial for ensuring balanced and efficient operation, particularly when operating in island mode or during dynamic load changes. The ability to share power proportionally among inverters is achieved through specific control mechanisms based on the droop controller. This ensures that each inverter contributes according to its rated capacity while maintaining system stability and reliability.

Furthermore, an adaptive centralized secondary control is implemented to restore frequency and voltage deviations caused by primary control. In this approach, a central controller manages setpoints for all resources, providing precise regulation; however, it may introduce latency and pose single-point-of-failure risks. Additionally, it helps maintain system stability under changing conditions. BESS commonly utilizes lithium-ion batteries due to their high energy density and efficiency, making them suitable for short- to medium-term storage applications such as peak shaving and load leveling. The proposed work in this paper utilizes the lithium-ion battery based on an effective control technique in order to preserve the DC bus voltage approximately constant during various events and to achieve the balance between the load and generation. The simple-to-implement control schemes for the battery are highly efficient in managing intermittent output power from PV units. Furthermore, the proposed control method, which integrates both the battery and PV system, achieves the main objective without relying on an ideal DC source, offering a more realistic and robust solution for real-time simulation.

The main contributions of this work are summarized as follows:

- **Primary Control Implementation**: Utilizes droop control to regulate voltage and frequency in a decentralized manner.
- **Adaptive Centralized Secondary Control**: Implements adaptive centralized control to restore frequency and voltage deviations caused by load variations and intermittent PV, providing accurate setpoints for voltage and frequency.
- **BESS Integration for Voltage and Load Balancing**: Integrates a lithium-ion BESS for voltage stabilization and load balancing. The BESS mitigates DC bus voltage instabilities, reduces fluctuations from PV intermittency, and supports MG stability under variable conditions.
- **PV-BESS integration**: Models PV as a real energy source with actual fluctuations, capturing weather variations and intermittent generation. This approach integrates PV and BESS into a unified control strategy, offering a more accurate and practical simulation for MG deployments.

This paper is structured as follows. Section “Introduction” introduction and literature review provide an overview of the research background, highlight key challenges, and summarize relevant studies in the field and the main contributions of this study. Section “System description” system description details the architecture, components, and operational framework of the proposed system, offering a comprehensive understanding of its design. Section “MG control approaches” control approaches explore various control strategies implemented to optimize system performance, enhance stability, and ensure efficient operation. Section “Simulation results and discussion” simulation results and discussion present the outcomes of simulations, critically analyze their implications, and evaluate the effectiveness of the proposed approach through comparative assessments. Section “Conclusions” the conclusion summarizes the key findings of the proposed study.

## System description

**Fig. 1:**
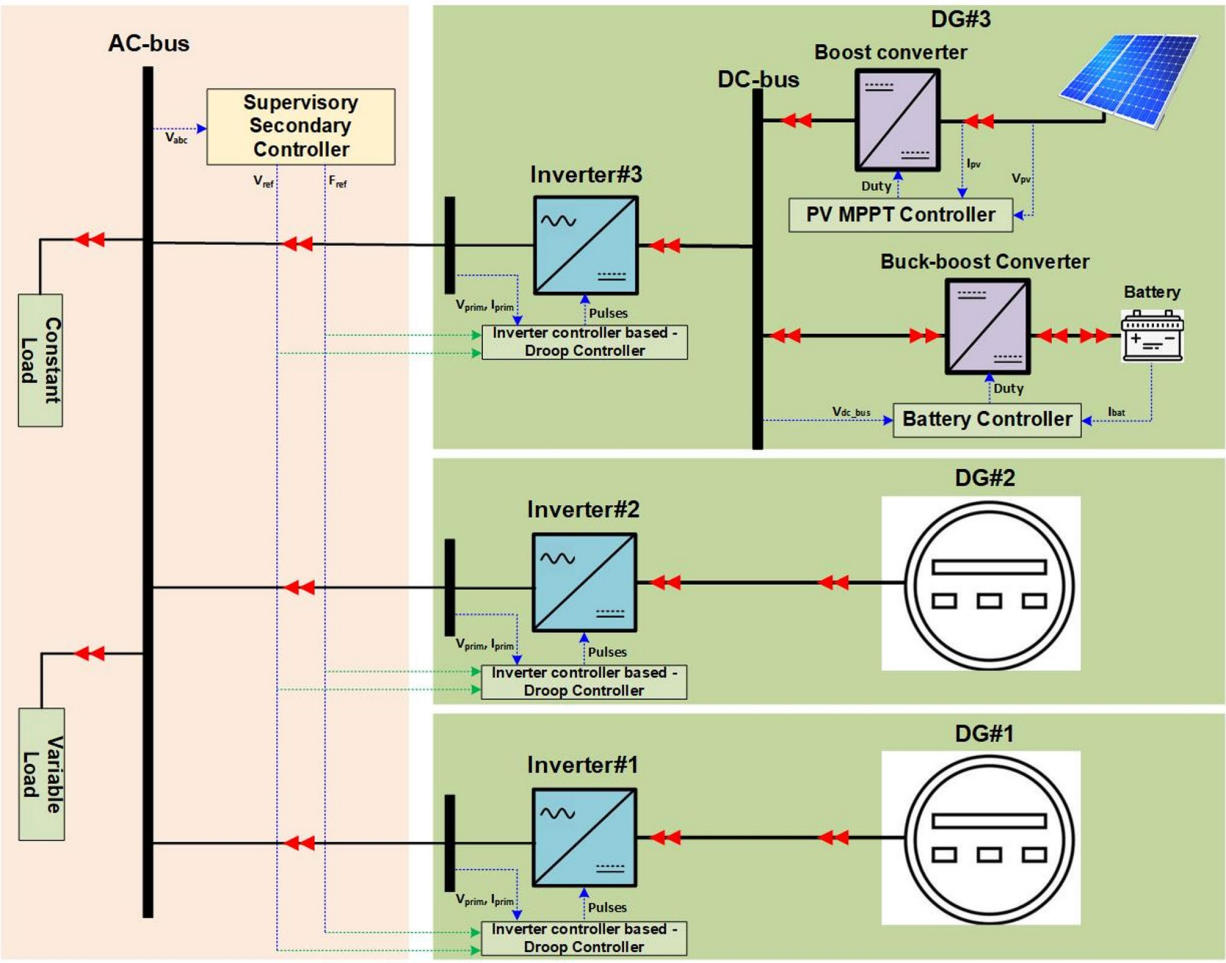
The overall proposed system structure.

The studied system is presented in Fig. 1 (the overall system), for which the original data is given in^24^. In this work, we have modified the original MG by incorporating PV and BSS systems (DG#3). It is simulated and relies on the MATLAB/Simulink software. This figure represents an MG system integrating DG units with a coordinated control structure. This MG consists of an AC bus as a central bus connecting all inverters and loads and supplying power to constant and variable loads. Moreover, three inverters (Inverter #1, Inverter #2, and Inverter #3) interface the DG units with the AC bus. Each inverter uses a droop controller for decentralized power sharing based on voltage (*V*_*prime*_) and current (*I*_*prime*_) feedback. Pulse signals are generated to control the inverter operation. Also, it has DGs #1 and #2, which are considered ideal DC sources connected to Inverter #1 and Inverter #2, respectively.

DG#3 is considered two units from a PV system with BSS connected to Inverter #3. PV is based on MPPT (Maximum Power Point Tracking) and BSS, which is connected via a buck-boost converter for charging/discharging and managed by a battery controller monitoring. Finally, the supervisory secondary controller monitors and adjusts the overall system performance. Table 2 lists all system component parameters.

**Table 2:** The nominal parameters of the studied system.

Parameter	Symbol	Unit	Value
Sampling time	$\:{T}_{s}$	$\:{\upmu\:}\text{s}$	0.5
line-line RMS voltage	V	$\:\text{V}$	$\:600$
Frequency	F	Hz	60
DC-bus reference voltage	*V*_*dc_ref_*_	V	1000
PV system			
Open-circuit voltage	V_oc_	V	321
Short-circuit current	I_sc_	A	393.36
Max. power point voltage	V_mp_	V	273.5
Max. power point current	I_mp_	A	368.28
Parallel strings	*Nser*	-	5
Series modules/string	*Npar*	-	66
Inductance	*L*_*PV*_	$\:\text{m}\text{H}$	5
Capacitance	*C*	mF	0.4
Battery			
Battery type	Lithium-ion		
Rated capacity	Ah	Ah	800
Capacitance	*C*	mF	1
Inductance	*L*_*bat*_	$\:\text{m}\text{H}$	1.5

## MG control approaches

The control approaches developed in this study are crucial to MG performance. The control scheme for each component is explained in detail below.

### PV system control

**Fig. 2:**
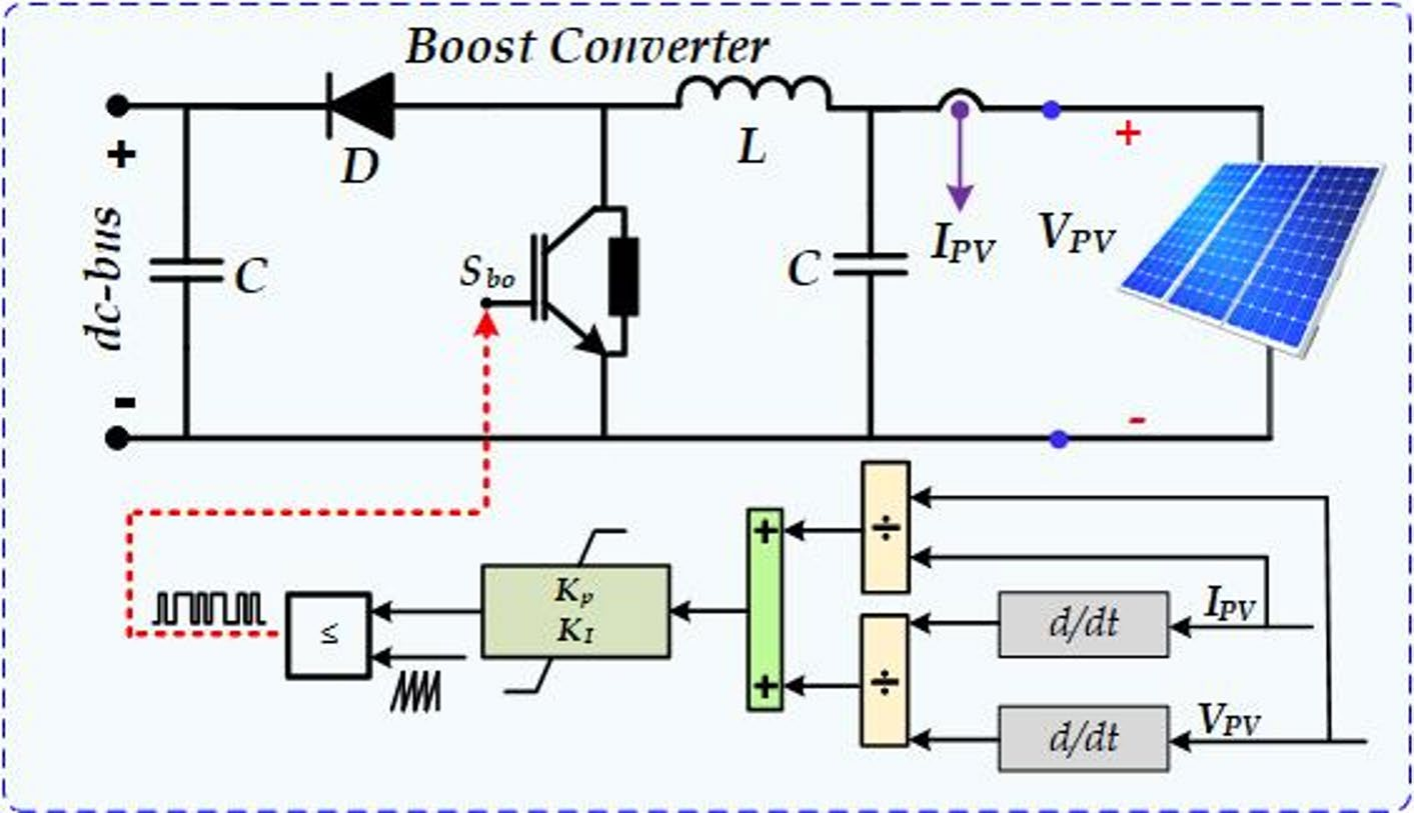
The MPPT controls the PV system.

Figure 2 illustrates a boost converter system integrated with an MPPT controller for a PV solar panel. The converter, connected to the PV array, increases the generated voltage to match the inverter’s high DC bus voltage. Specifically, it boosts the array’s DC voltage using the Incremental Conductance (INC) method, which adjusts the switching duty cycle. The duty cycle setpoint is automatically regulated by the MPPT-based INC technique to achieve the desired voltage, ensuring maximum power extraction from the PV array^25^. The INC method accurately tracks the PV module’s peak output and improves dynamic performance, particularly under rapidly changing environmental conditions. The algorithm first measures the voltage and current at the PV array terminals, and then the INC approach measures the reference value for the inductor current based on the variation in voltage and power relative to the prior stage. This incremental change is applied to update the reference value of the inductor current at each step, striking a balance between maximizing MPPT tracking speed and minimizing power oscillations. Equations (1) and (2) present the operation of the INC method applying the ideal P–V curve of a standard PV unit^25^. When Eq. (1) is achieved, the PV unit operates at the MPP on the P–V curve, with corresponding optimal values for current and voltage. If the current and voltage deviate from their optimal values, the effective controller resets the incremental values to assist the system in following the MPP approach. The MPPT controller dynamically modifs the boost converter’s duty cycle to achieve and maintain MPP operation.

1 $$\frac{{d{P_{PV}}}}{{d{V_{PV}}}}=0 \to {I_{PV}}+{V_{PV}}\frac{{d{I_{PV}}}}{{d{V_{PV}}}}=0~~~$$.

2 $$\frac{{d{P_{PV}}}}{{d{V_{PV}}}}= - \frac{{d{I_{PV}}}}{{d{V_{PV}}}}~~$$

### Battery control system

Maintaining a stable DC bus voltage is crucial, as fluctuations can directly impact the load voltage. Therefore, ensuring a constant DC bus voltage is essential for reliable system performance. The battery control system plays a vital role in this process. In addition, the battery helps regulate power flow during abnormal operating conditions or when power generation from the PV system fluctuates. A typical battery model consists of an open-circuit voltage (OCV) source, internal resistance, and dynamic components that represent the battery’s charge and discharge behavior. The most commonly used equation describing this behavior is presented in Eq. (3)^26–28^. The state of charge (SoC) is defined by Eqs. (4) and (5).

3 $${V_{bat}}={V_{oc}} - {I_{bat}}{R_{int}}$$

Where:

*V*_*bat*_ = battery terminal voltage (V). *V*_*oc*_ = open-circuit voltage (V), which depends on the state of charge (SoC). *I*_*bat*_ = battery current (A). *R*_*int*_ = internal resistance (Ω).

4 $$SoC~\left( t \right)=SoC~\left( 0 \right) - \frac{1}{{{C_{bat}}}}\mathop \smallint \limits_{0}^{t} {I_{bat}}\left( \tau \right)d\tau$$

5 $$SoC~\left( k \right)=SoC~\left( {k - 1} \right) - \frac{{\Delta t}}{{{C_{bat}}}}{I_{bat}}\left( k \right)$$

Where:

$SoC~\left( 0 \right)$ = initial SoC *C*_*bat*_ = battery capacity (Ah). *Δt* = time step (s).

**Fig. 3:**
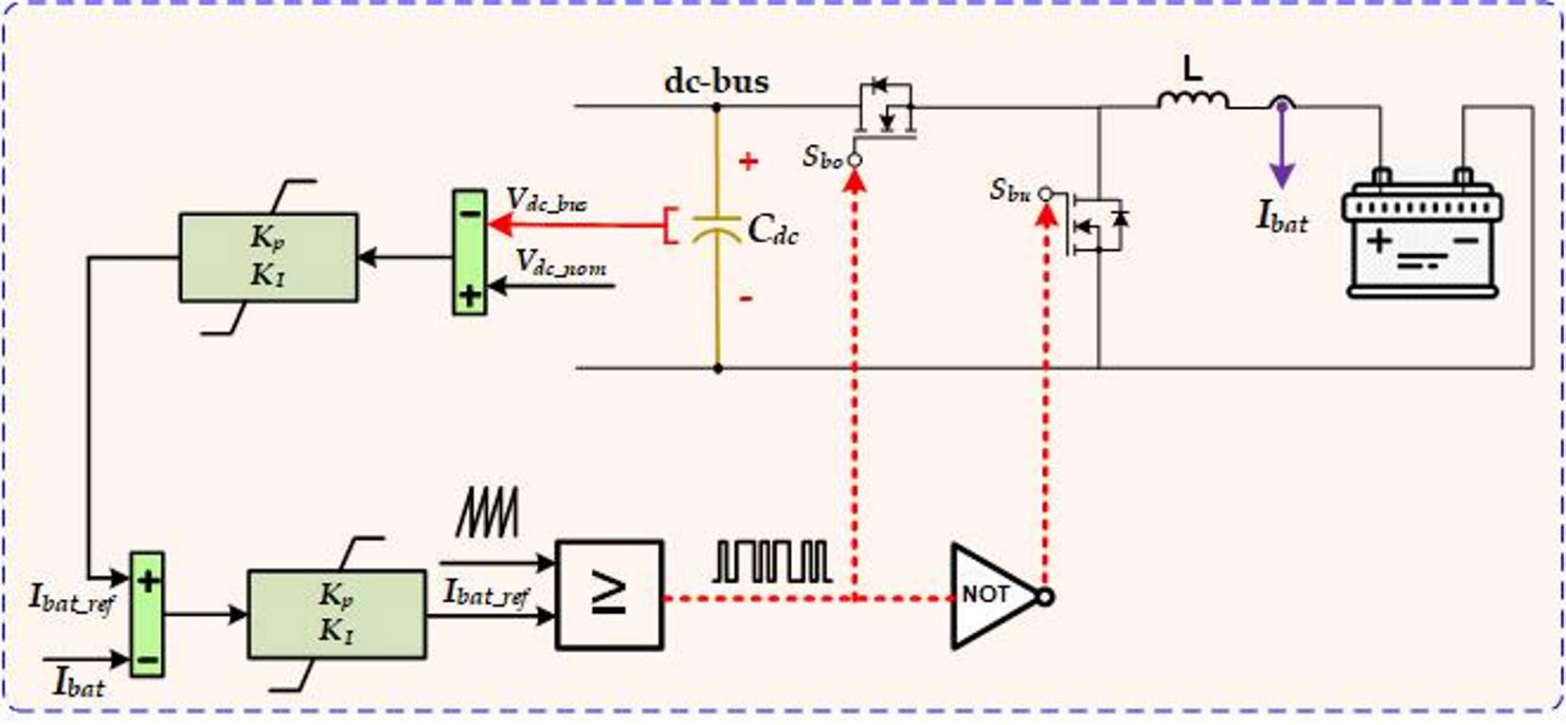
The control of the battery system.

The battery’s operation is triggered by the difference between the actual DC bus voltage and its reference value. Charging Mode: when *V*_*dc*_ exceeds *V*_*dc_nom*_, the PI controller reduces the DC-bus voltage by charging the battery through *S*_*bo*_. Discharging Mode: when *V*_*dc*_ falls below *V*_*dc_nom*_, the battery discharges through *S*_*bu*_ to supply power to the DC-bus, stabilizing the voltage. The battery control strategy is shown in Fig. 3, where the actual battery current is compared to the reference current generated by the previous control loop using PI controllers. This control strategy enhances the stability and reliability of power systems, particularly in setups with renewable energy sources.

### The primary and secondary control of MG

**Fig. 4:**
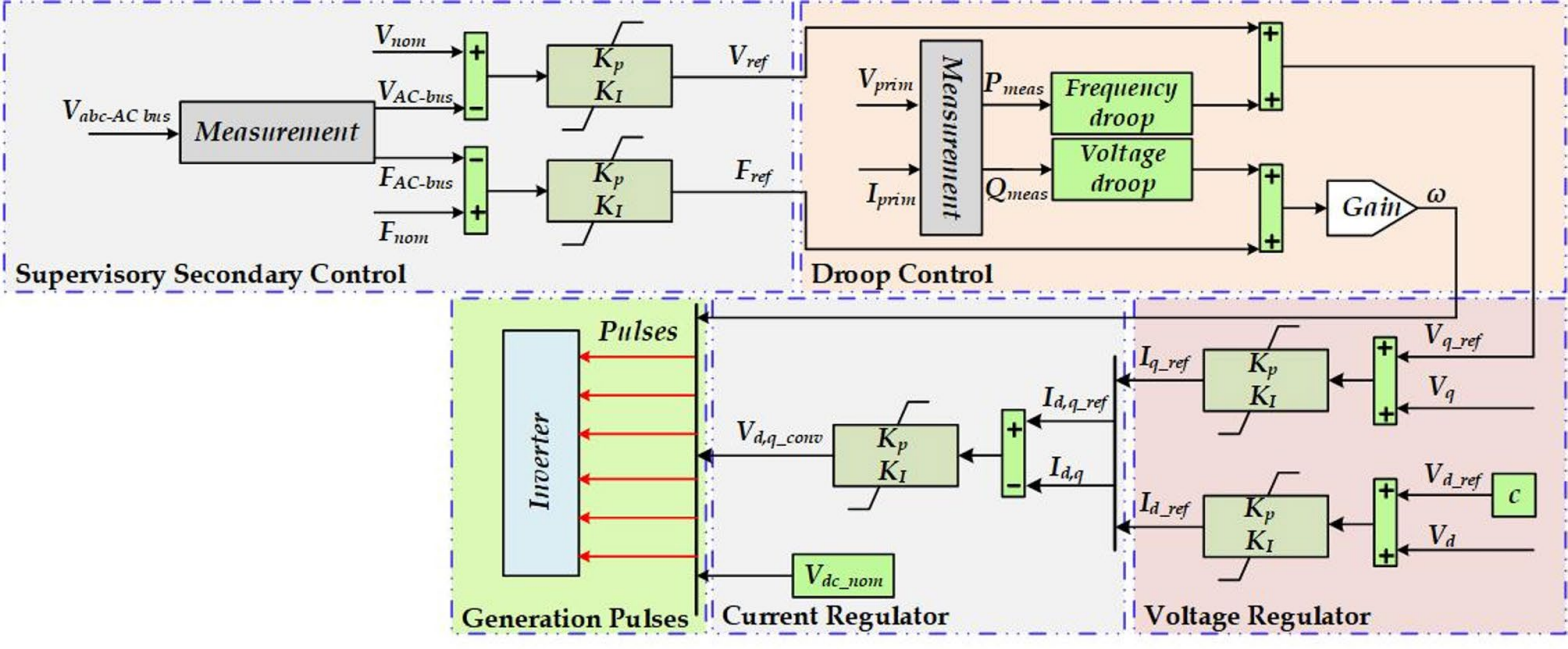
The primary and secondary control of MG.

Figure 4 illustrates a hierarchical control structure for a MG system, incorporating supervisory secondary control, droop control, current regulation, and voltage regulation. Each control layer plays a role in maintaining system stability, ensuring proper power sharing, and regulating voltage and frequency in the MG. *P-F* droop control equations are presented in (6) and (7)^29,30^, where this control method adjusts the frequency in response to active power changes. Meanwhile, the Q-V droop control equations are presented in (8) and (9)^29,30^, where the method regulates voltage based on reactive power demand.

The supervisory secondary control receives inputs from *V*_*abc−AC*__bus, representing the three-phase voltage measurements, *V*_*AC−bus*_, the RMS voltage of the AC bus, and *F*_*AC−bus*_, the bus frequency.

These measurements are compared with nominal setpoints *V*_*nom*_ and *F*_*nom*_, and two PI controllers generate *V*_*ref*_ (voltage reference) and *F*_*ref*_ (frequency reference) to maintain the desired operating conditions by adjusting droop parameters. The droop control, with inputs *V*_*prim*_ and *I*_*prim*_, calculates active power *P*_*meas*_ and reactive power *Q*_*meas*_, adjusting frequency through frequency droop and voltage via voltage droop mechanisms to enable decentralized load sharing among inverters without direct communication. The inverter, receiving control signals, generates PWM pulses to regulate DC-to-AC conversion and manage power delivery to the grid or loads. The current regulator, utilizing *V*_*dq_conv*_ (converter voltage in the dq-axis reference frame) and *V*_*dc_nom*_ (nominal DC voltage), employs a PI controller to produce *I*_*dq−ref*_, ensuring that *d-axis* and *q-axis* currents (*I*_*dq*_) control active and reactive power effectively. Subsequently, the voltage regulator receives *I*_*dq−ref*_ and uses separate PI controllers to regulate *V*_*q*_ and *V*_*d*_ against their respective references *V*_*q−ref*_ and *V*_*d−ref*_, thereby maintaining the inverter output voltage at the desired levels. The coordinated operation of these control layers ensures the MG operates at stable voltage and frequency, allows decentralized power sharing, maintains power quality, and enhances the flexibility and resilience of the overall system.

6 $$f={f_0} - {m_p}\left( {P - {P_0}} \right)$$

7 $$\Delta f= - {m_p}\Delta P$$

where:

*f*: operating frequency (Hz). *f*_*0*_ : nominal frequency (Hz). *P*: actual active power output (W). *P*_*0*_ : nominal active power (W). *m*_*P*_: droop coefficient (Hz/W). $\Delta f:$ the difference between operating frequency and nominal frequency. $\Delta P:$ the difference between actual active power and nominal active power.

8 $$V={V_0} - {m_Q}\left( {Q - {Q_0}} \right)$$

9 $$\Delta V= - {m_Q}\Delta Q$$

where:

*V*: operating voltage (V). *V*_*0*_: nominal voltage (V). *Q*: actual reactive power (VAR). *Q*_*0*_: nominal reactive power (VAR). *m*_*Q*_: droop coefficient (V/VAR). $\Delta V$: difference between operating voltage and nominal voltage. $\Delta Q$: difference between actual reactive power and nominal reactive power.

## Simulation results and discussion

The studied system presented in Fig. 1 is simulated and relied on the MATLAB/Simulink software. The simulation results comprise four different cases to indicate the performance of the proposed method. The studied system consists of three DG units; two units are simulated through ideal DC sources with two converters; meanwhile, the third one (the proposed method) is based on using the batteries and PV systems with a PI controller instead of using an ideal DC source. Different cases are performed with variable load conditions to examine the proposed control method.

### Case 1

**Fig. 5:**
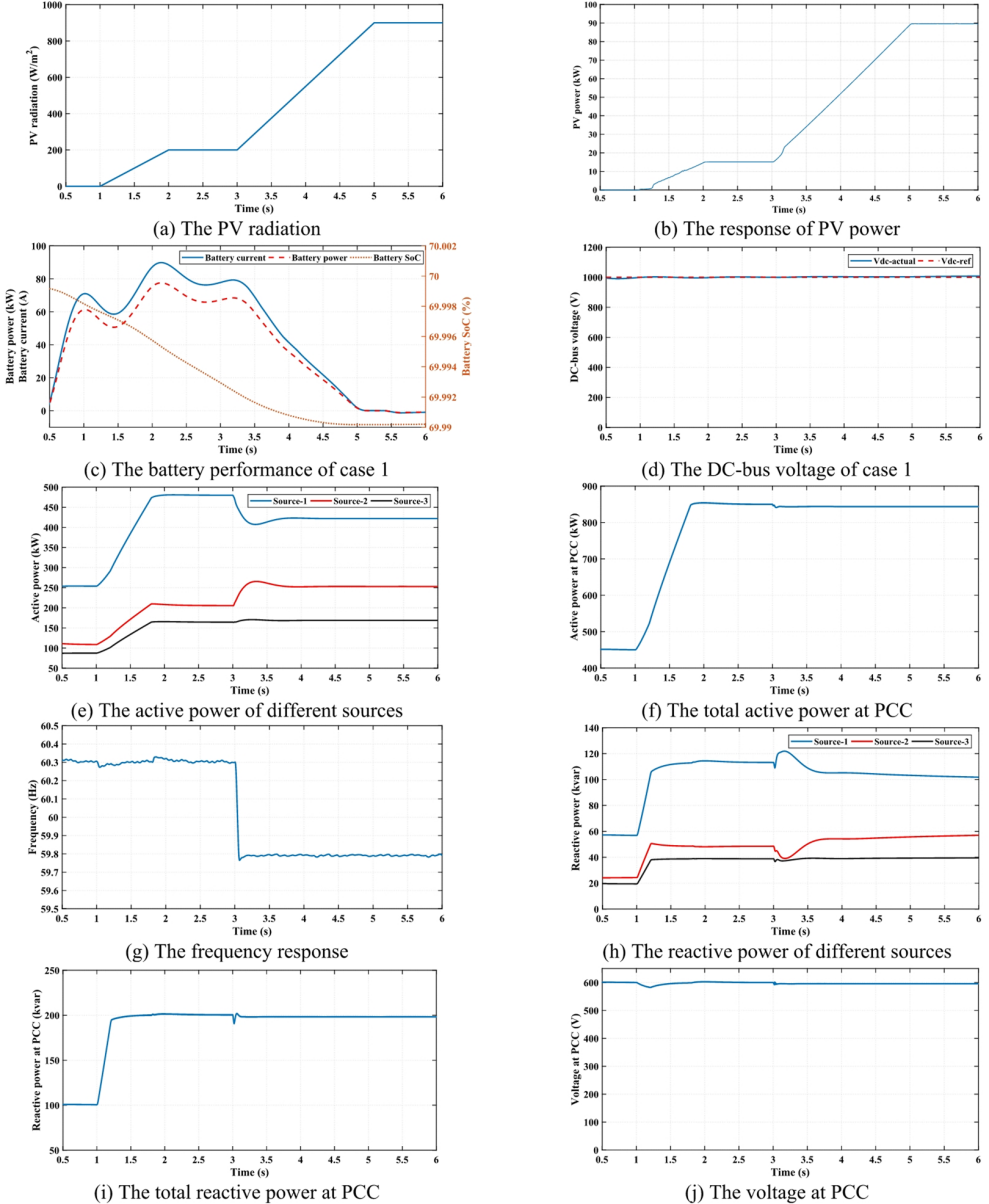
The simulation results of case 1.

In this case, the proposed control method is evaluated under varying PV radiation conditions, as depicted in Fig. 5 (a). Figure 5 (b) illustrates the PV system’s corresponding power output during this scenario. The PV farm consists of two independent PV systems, and it is evident that the generated PV power exhibits random fluctuations due to variations in environmental conditions. These fluctuations directly impact the system frequency, leading to instability. A BESS is integrated into the system to mitigate frequency deviations caused by these climatic changes. BES operational performances, specifically battery current, SoC, and power, are presented in Fig. 5 (c). The batteries dynamically regulate their charging and discharging processes based on the MG’s instantaneous power requirements, ensuring stable operation. The effectiveness of the MPPT control strategy is demonstrated by its ability to extract the maximum available power from the PV, as validated by the PV power response. In addition to optimizing power extraction, a PI control approach is implemented for the battery system to maintain a stable DC bus voltage. As shown in Fig. 5 (d), this control mechanism ensures that the DC voltage remains approximately constant, allowing the battery to function as a stable DC power source. The combined use of MPPT and PI control enhances system stability, improves energy utilization efficiency, and ensures smooth delivery under dynamic environmental conditions.

The regulation of the DC bus voltage ensures that its behavior closely follows that of an ideal DC source, maintaining stability and enhancing the overall performance of the MG system. This voltage stabilization plays a crucial role in ensuring seamless power integration from different energy sources. Figure 5 (e) illustrates the dynamic power-sharing mechanism among all system components, including the PV and BESS as source 3. The power-sharing strategy efficiently distributes the generated power that is based on the active power-frequency (P-f) droop characteristic that is applied at 3*s*, ensuring that generators reduce or increase power output based on frequency variations; in turn, this leads to the MG operating within its designed constraints while maintaining optimal performance. Meanwhile, Fig. 5 (f) presents the total power at the PCC, highlighting the combined contribution of the PV system, battery storage, and other distributed energy resources. The total power at the PCC reflects the compensatory response of the battery system to overcome the intermittent nature of the solar irradiance.

The impact of variable solar radiation on system frequency is analyzed in Fig. 5 (g), which demonstrates the effectiveness of the battery system with its controller to be an ideal DC source and the droop controller, which is applied at 3*s*. These frequency variations necessitate effective frequency regulation strategies to ensure system stability and reliability. The BSS plays a vital role in mitigating frequency deviations by dynamically adjusting its charging and discharging states to counteract the variations in PV output and maintain the DC voltage at a constant value. In addition to active power regulation, reactive power compensation is also a crucial aspect of MG stability. The three inverters-based reactive power-voltage (Q-V) droop characteristic helps maintain voltage levels and ensures efficient power flow within the system by adjusting reactive power output based on voltage variations within the system, as shown in Fig. 5 (h).

Moreover, Fig. 5 (i) shows the reactive power injected into the MG, presenting the contribution of three DGs in the reactive power support, which is essential for voltage regulation, especially under changing load demands and varying PV radiation conditions. As depicted in Fig. 5 (j), the voltage at the PCC remains approximately constant which shows minimal deviations in PCC voltage despite dynamic operating conditions. The ability of the system to maintain a stable voltage profile under variable environmental and load conditions highlights the effectiveness of the proposed control strategy in ensuring the reliable and efficient operation of MG. The integrated approach of reactive and active power management, linked with effective frequency and voltage regulation, makes it well-suited for real applications where RES integration is essential, moreover, to improve the overall modern power system robustness.

### Case 2

**Fig. 6:**
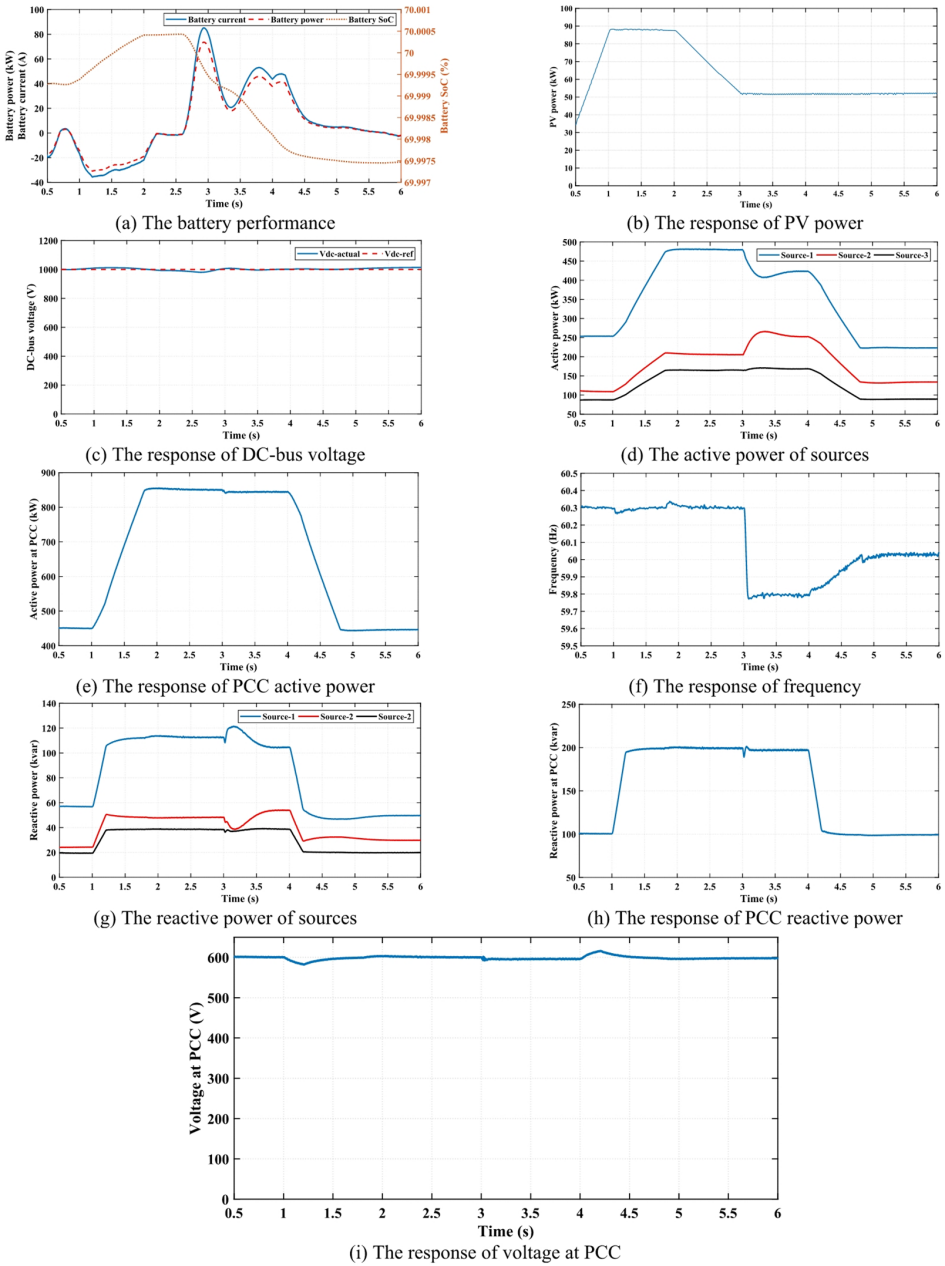
The simulation results of case 2.

The proposed control technique is utilized to manage power exchange between the MG and the battery, taking into account the conditions of dynamic operation. The performance of the battery is illustrated in Fig. 6 (a), which shows the charging and discharging process through key parameters such as battery current, power, and SoC. When the sign of power is positive, this shows the discharging process of the battery and provides active power into the MG to support the system during periods of high demand or low PV output power. On the other hand, when the sign of power is negative, that indicates the battery is charging, absorbing excess energy from the MG to store for peak periods and high load demand. The control technique proves that the battery operates efficiently by responding dynamically to fluctuations in load demand and intermittent PV output power.

The robustness and effectiveness of the proposed control approach are tested with the load variation process where it is injected at 1s and rejected at 4s, with intermittent power generation from PV units, as presented in Fig. 6 (b). The battery regulates these variations by adjusting its charging and discharging states accordingly. This dynamic regulation helps maintain system stability and ensures a continuous and reliable power supply which emphasizes the essential role of BESS in MG operation.

One of the most significant conditions for MG stability performance is sustaining the value of DC voltage approximately constant during the sever events such as PV power and load variation. The proposed control approach effectively regulates the DC bus voltage, keeping it within acceptable limits, as demonstrated in Fig. 6 (c); the DC bus voltage remains stable during load insertion, load rejection, and fluctuations in PV radiation. The ability to sustain a nearly constant DC voltage ensures that connected loads receive a steady power supply, preventing undesirable voltage deviations that could lead to system instability.

The proposed method effectively manages the power-sharing dynamics among the three sources, as illustrated in Fig. 6 (d). The total active power at the PCC is depicted in Fig. 6 (e), highlighting the load insertion event at 1*s* and the load rejection at 4*s*. These changes in load, along with variations in PV radiation, lead to fluctuations in frequency response, as detailed in Fig. 6 (f). In addition to supplying active power, the three available sources contribute reactive power to the MG, as demonstrated in Fig. 6 (g). The total reactive power at the PCC is presented in Fig. 6 (h), showing that the proposed method successfully enhances MG performance by ensuring adequate reactive power support to system components. This improvement in reactive power management positively impacts voltage stability at the PCC, as evidenced by the results in Fig. 6 (i).

### Case 3

**Fig. 7:**
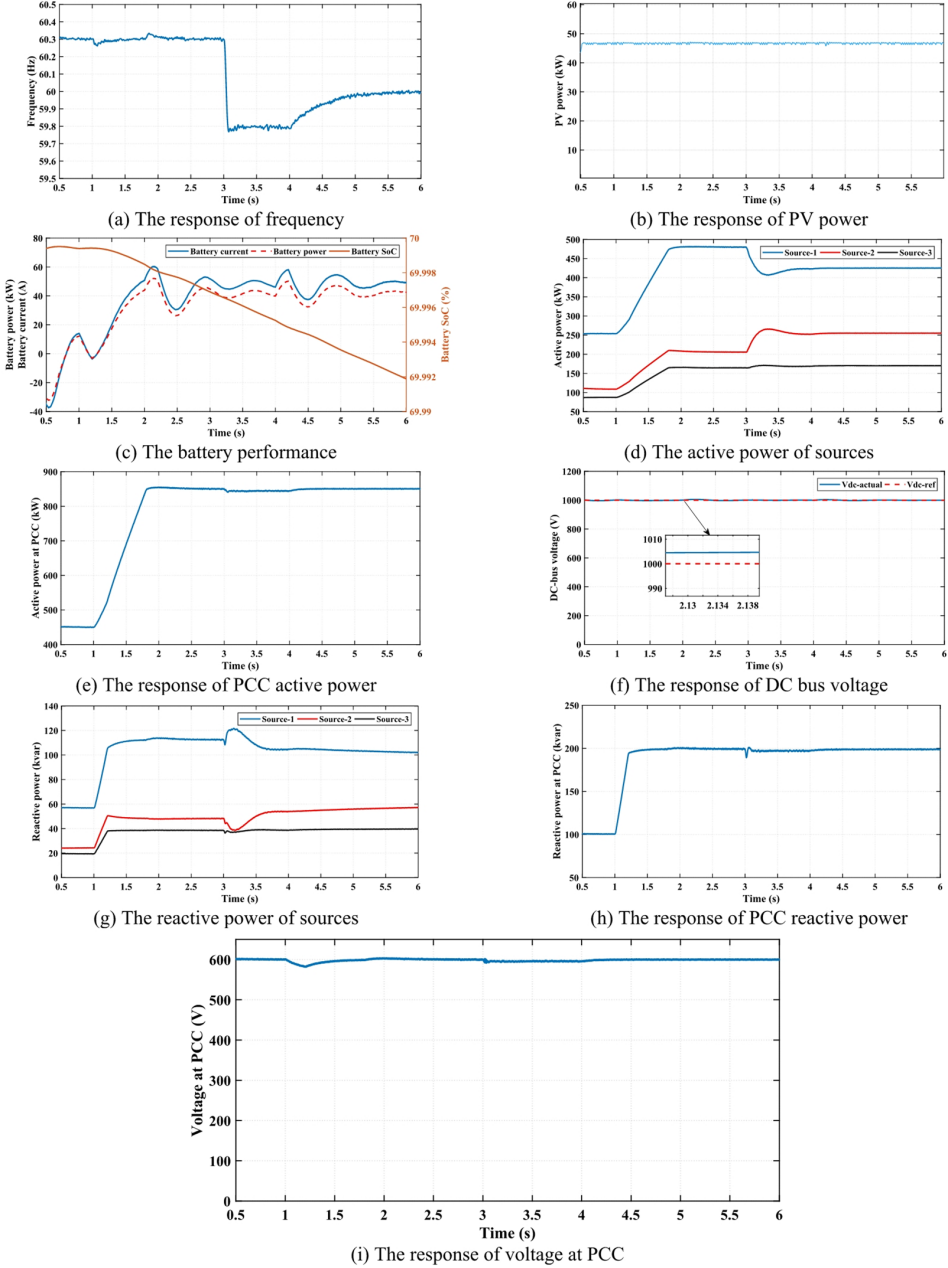
The simulation results of case 3.

In this scenario, the active and reactive power set-points are dynamically adjusted to enhance system frequency and voltage stability during load variations. This is achieved using an adaptive centralized secondary control method, which actively regulates power distribution among the available sources. The frequency response is effectively restored to the desired nominal value by modifying the active power set-point, as illustrated in Fig. 7 (a). To isolate the impact of the active power set-point adjustment, PV radiation is assumed to remain constant, ensuring a precise analysis of the control method’s effect, as shown in Fig. 7 (b). Since Source 3 consists of a battery integrated with a PV system, its performance is analyzed in Fig. 7 (c), which presents key battery parameters such as SoC, battery current, and battery power. Under the proposed control strategy, the battery system plays a crucial role in maintaining system performance by ensuring the continuous availability of active power. This capability is particularly important during periods of reduced PV power generation, as demonstrated in Fig. 7 (d). Meanwhile, the total power injected into the loads at the PCC is depicted in Fig. 7 (e).

The effectiveness of the control method for coordinating the battery and PV system is further validated by monitoring the DC bus voltage, which serves as the common connection point for both energy sources. Maintaining a stable DC bus voltage is critical for ensuring seamless power exchange between the battery, PV system, and MG, and the proposed method successfully regulates it under varying operational conditions, as indicated in Fig. 7 (f). Beyond frequency stabilization, the three sources collectively contribute to meeting the reactive power demand of the MG. The reactive power supplied to the system components enhances voltage stability, as shown in Fig. 7 (g). Meanwhile, the total reactive power delivered to the loads at the PCC is presented in Fig. 7 (h). By ensuring adequate reactive power support, the proposed control strategy significantly improves the voltage profile at the PCC, as evidenced in Fig. 7 (i). These results confirm the effectiveness of the adaptive centralized secondary control method in optimizing MG performance under dynamic conditions.

### Case 4

In this case, a representative sample of the obtained results is presented to validate the effectiveness of the proposed method. This validation is achieved by comparing the performance of the proposed control strategy. Source 3 consists of a battery and a PV system with a dedicated controller. In the conventional case, sources 1, 2 and 3 operate as ideal DC sources, in which all three sources function as ideal DC sources without additional control.

**Fig. 8:**
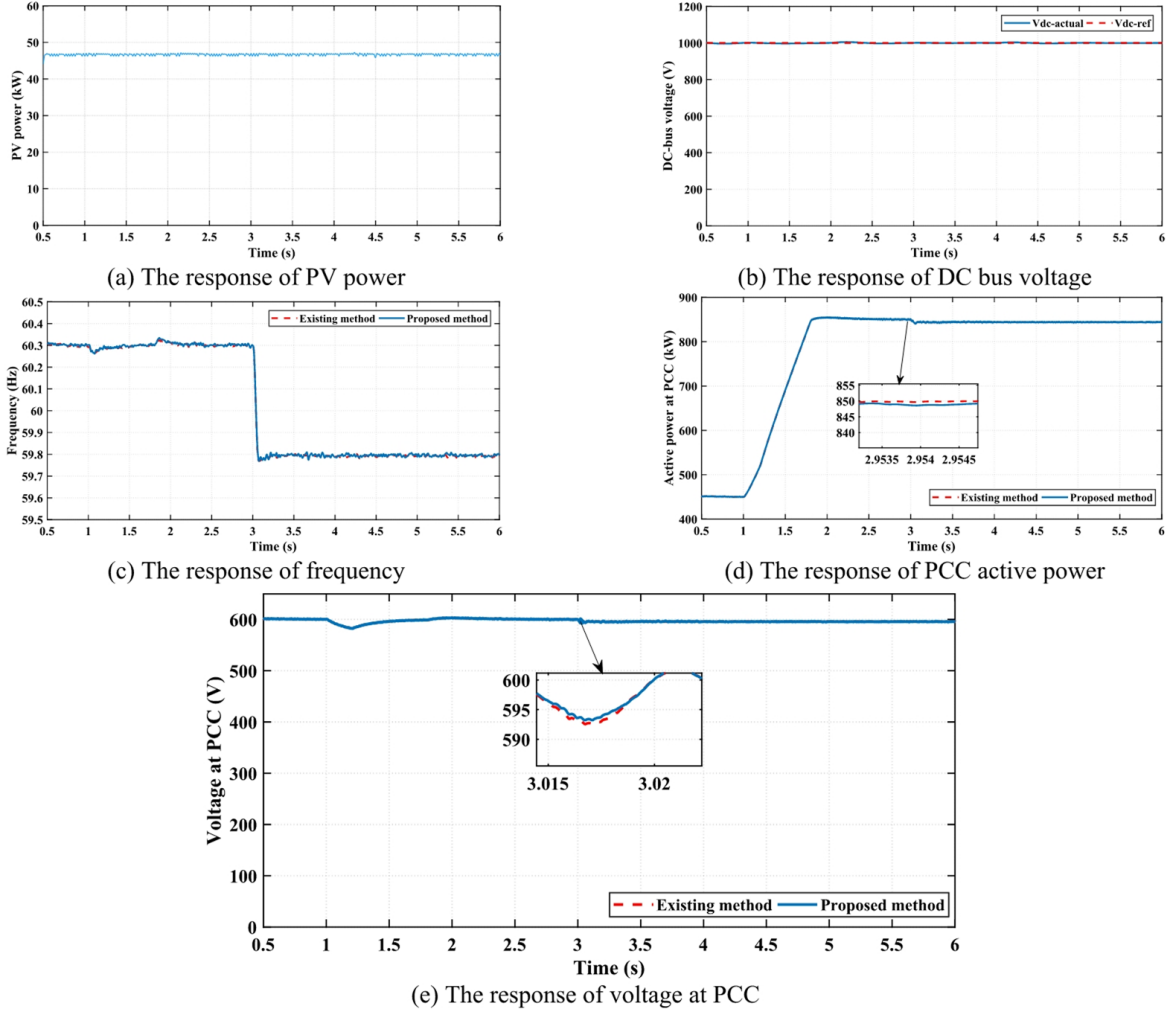
The simulation results of case 4.

The dynamic response of the PV active power contribution under the proposed method is illustrated in Fig. 8 (a), highlighting its role in the overall power management strategy. Additionally, the stability and regulation of the DC bus voltage, a critical parameter for ensuring the proper coordination between the PV and battery system, are demonstrated in Fig. 8 (b). To further assess the effectiveness of the proposed method, the system frequency response is analyzed under two scenarios: one employing the battery and PV system and the other utilizing an ideal DC source-based model. The comparison, as depicted in Fig. 8 (c), reveals the similarity in frequency stabilization between the two approaches, demonstrating the robustness of the proposed control method.

Figure 8 (d) shows the comparison between the proposed method and the existing method in terms of active power absorbed by the loads at the PCC, which proves the effectiveness of the proposed control method to ensure reliable and efficient power delivery, even when incorporating the battery and PV system as a controllable energy source. Finally, the voltage response at the PCC is examined in both scenarios, as illustrated in Fig. 8 (e). The almost identical between the proposed and existing methods of voltage response validates that the integration of battery and PV sources does not negatively impact system performance. As a comprehensive comparison between all case studies, Table 3 lists a summary and comparison of different case studies. These findings collectively demonstrate the capability of the proposed control strategy to enhance MG stability and power-sharing efficiency while maintaining performance comparable to the existing method. Finally, Table 4 summarizes the advantages and limitations of the proposed approach, highlighting the use cases and typical limitations.

**Table 3:** Summarize and comparison of different case studies.

Feature	Case 1	Case 2	Case 3	Case 4 (for comparison)	
	Proposed	Proposed	Proposed	Proposed	Existing
Droop control	ON → 3 *s*	ON → 3 *s*	ON → 3 *s*	ON → 3 *s*	ON → 3 *s*
Supervisory secondary control	OFF	OFF	ON → 4 *s*	OFF	OFF
Variable load	ON → 1 *s*	ON → 1 *s* OFF → 4 *s*	ON → 1 *s*	ON → 1 *s*	ON → 1 *s*
PV power	Yes→Variable	Yes→Variable	Yes→Constant	Yes→Constant	NO
Battery	Yes→ It can charge/discharge based on the control process	Yes→ It can charge/discharge based on the control process	Yes→ It can charge/discharge based on the control process	Yes→ It can charge/discharge based on the control process	NO
Main achievements and targets	Frequency (from 0.5 *s* to 3 *s* → 60.3 Hz, from 3 *s* to 6 *s* → 59.8 Hz) PCC voltage→ approximately constant at 600 V DC bus voltage→ approximately constant at 1000 V Power sharing between different DG sources→ Achieved	Frequency (from 0.5 *s* to 3 *s* → 60.3 Hz, from 3 s to 4 *s* → 59.8 Hz, from 4 *s* to 6 *s* → 60.05 Hz) PCC voltage→ approximately constant at 600 V DC bus voltage→ approximately constant at 1000 V Power sharing between different DG sources→ Achieved	Frequency (from 0.5 *s* to 3 *s* → 60.3 Hz, from 3 s to 4 *s* → 59.8 Hz, from 4 *s* to 6 *s* → 60 Hz) PCC voltage→ approximately constant at 600 V DC bus voltage→ approximately constant at 1000 V Power sharing between different DG sources→ Achieved	Frequency (from 0.5 *s* to 3 *s* → 60.3 Hz, from 3 *s* to 6 *s* → 59.8 Hz) PCC voltage→ approximately constant at 600 V DC bus voltage→ approximately constant at 1000 V Power sharing between different DG sources→ Achieved	Frequency (from 0.5 *s* to 3 *s* → 60.3 Hz, from 3 *s* to 6 *s* → 59.8 Hz) PCC voltage→ constant at 600 V DC bus voltage→ constant at 1000 V Power sharing between different DG sources→ Achieved

**Table 4:** Summarizes the advantages and limitations of the proposed approach.

Approach	Pros	Cons	Typical limits	Purpose
Droop + Supervisory secondary control + BESS	Local sharing; droop; supervisory secondary control removes steady-state error; realistic PV + MPPT; strong DC-bus hold	Supervisory secondary control adds a Single Point of Failure risk due to a communication issue	Needs coordinated tuning (PI vs. $\:{m}_{P}/{m}_{Q}$); reliable measurements/ comms	Mixed PV + BESS MGs needing resilience and accuracy

## Conclusions

This study introduces a comprehensive control strategy to enhance MG stability, effectively addressing critical challenges such as power-sharing, frequency regulation, voltage stabilization, and intermittent power of RES. The proposed framework, comprising droop control, adaptive centralized secondary control, and BESS integration, demonstrates robust performance under dynamic load and environmental conditions. The obtained simulation results based on MATLAB software validate the robustness and effectiveness of the control layers: droop-based primary control enables decentralized operation and proportional power-sharing, while the adaptive centralized secondary control set and restores voltage and frequency to nominal values. The integration of BESS enhances system stability by regulating the DC bus voltage and balancing power flow, particularly during fluctuations in PV output power. The use of a PV model, as opposed to an idealized DC source, increases the realism and practical relevance of the study, providing more accurate insight into actual MG behavior. Comparative analysis with the existing system shows that the proposed method achieves comparable voltage and frequency stabilization while offering superior adaptability to changing conditions. This ability to manage power fluctuations, maintain system stability, and ensure efficient power-sharing underscores the potential of the proposed strategy for future applications of MG. Overall, the findings support the development of robust, efficient, and scalable control methods for MGs powered by RESs.

Future research may explore the integration of advanced machine learning algorithms to enable predictive maintenance and optimize the operations of MG. Using real-time data and predictive analytics, these approaches could enhance the responsiveness and intelligence of the control framework, especially in handling unexpected variations in RESs and demand. Furthermore, assessing the scalability of the proposed strategy in larger, more complex MG networks and examining its interoperability with modern power systems would further support its practical deployment. Experimental validation across diverse environmental conditions and RESs will also be crucial in reinforcing the robustness and adaptability of the control strategy, enabling practical deployment.

## Data Availability

All data generated or analysed during this study are included in this published article.

## References

[1] Uddin, M. et al. *Microgrids: A Review, Outstanding Issues and Future Trends* (Elsevier Ltd., 2023). 10.1016/j.esr.2023.101127.

[2] Saadati, T. et al. Enhancing microgrid voltage and frequency stability through multilayer interactive control framework. *Int. Trans. Electr. Energy Syst.* **1**, 4933861 (2024).

[3] Shahzad, S. et al. Possibilities, cchallenges, and future opportunities of microgrids. *Rev. Sustain.* **15** (8), 6366. 10.3390/su15086366 (2023).

[4] IEA. Share of renewable electricity generation by technology, 2000-2030, IEA, Paris. https://www.iea.org/data-and-statistics/charts/share-of-renewable-electricity-generation-by-technology-2000-2030, Licence: CC BY 4.0 (2024).

[5] Koohi-Fayegh, S. & Rosen, M. A review of energy storage types, applications and recent developments. *J. Energy Storage*. **27**, 101047. 10.1016/j.est.2019.101047 (2020).

[6] Georgious, R., Refaat, R., Garcia, J. & Daoud, A. A. Review on energy storage systems in microgrids. *Electronics* **10** (17), 2134. 10.3390/electronics10172134 (2021).

[7] Majeed, M. A., Phichaisawat, S., Asghar, F. & Hussan, U. High-efficiency renewable penetration via dynamic decentralized droop control in microgrid systems.* IEEE Access* **12**, 143500–143514. 10.1109/ACCESS.2024.3466187 (2024).

[8] Eyisi, C. & Li, Q. Analysis and optimization of boost converter parameters in internal model control for voltage source converter-Based AC microgrids. *CSEE J. Power Energy Syst.* **10** (5), 2038–2054. 10.17775/CSEEJPES.2022.06880 (2024).

[9] Pinthurat, W., Kongsuk, P., Surinkaew, T. & Marungsri, B. An adaptive data-driven-based control for voltage control loop of grid-forming converters in variable inertia MGs. *IEEE Access* **12**, 58143–58155. 10.1109/ACCESS.2024.3392295 (2024).

[10] Kumar, M. Development of control strategies for operation of cluster of interconnected hybrid microgrids in islanded mode. *IEEE Syst. J.* **17** (2), 1741–1752. 10.1109/JSYST.2023.3239738 (2023).

[11] García-Triviño, P. et al. Supervisory control system for a Grid-Connected MVDC microgrid based on Z-Source converters with PV, battery Storage, green hydrogen system and charging station of electric vehicles. *IEEE Trans. Ind. Appl.* **59** (2), 2650–2660. 10.1109/TIA.2022.3233556 (2023).

[12] Bevrani, H., Ise, T. & Miura, Y. Virtual synchronous generators: a sur-vey and new perspectives. *Int. J. Electr. Power Energy Syst.* **54**, 244–254 (2014).

[13] Tamrakar, U. et al. Virtual inertia: current trends and future directions. *Appl. Sci.* **7** (7), 654 (2017).

[14] Lu, L. & Cutululis, N. A. Virtual synchronous machine control for wind turbines: a review. *J. Phys. Conf.*, **1356**, 012028 (2019).

[15] Rosso, R., Wang, X., Liserre, M., Lu, X. & Engelken, S. Gridforming converters: control approaches, grid-synchronization, and future trends—a review. *IEEE Open. J. Ind. Appl.* **2**, 93–109 (2021).

[16] Rathnayake, D. B. et al. Grid forming inverter modeling, control, and applications. *IEEE Access* **9**, 114781–114807 (2021).

[17] Mahmoud, K., Astero, P., Peltoniemi, P. & Lehtonen, M. Promising grid-forming vsc control schemes toward sustainable power systems: comprehensive review and perspectives. *IEEE Access* **10**, 130024–130039. 10.1109/ACCESS.2022.3228742 (2022).

[18] Yang, Y., Enjeti, P., Blaabjerg, F. & Wang, H. Wide-Scale adoption of photovoltaic energy: grid code modifications are explored in the Danish context. *IEEE Ind. Appl. Mag*. **21** (5), 21–31. 10.1109/MIAS.2014.2345825 (2015).

[19] Luo, L. et al. Optimal scheduling of a renewable based microgrid considering photovoltaic system and battery energy storage under uncertainty. *J. Energy Storage*. **28**, 101306. 10.1016/j.est.2020.101306 (2020).

[20] Alshehri, J. & Khalid, M. Power quality improvement in microgrids under critical disturbances using an intelligent decoupled control strategy based on battery energy storage system. *IEEE Access* **7**, 147314–147326. 10.1109/ACCESS.2019.2946265 (2019).

[21] Zare Ghaleh Seyyedi, A., Gitizadeh, M., Fakhrooeian, M. & Jabareh Nasero, M. Achieving the goals of energy arbitrage, peak-shaving, and PV self-consumption using PV-BTM BESS microgrids coupled with a distribution network. *J. Energy Storage*. **112**, 115479. 10.1016/j.est.2025.115479 (2025).

[22] Bai, Y., Wang, J. & He, W. Energy arbitrage optimization of lithium-ion battery considering short-term revenue and long-term battery life loss. *Energy Rep.* **8**, 364–371. 10.1016/j.egyr.2022.10.209 (2022).

[23] De Souza Silva, J. L., Moreira, H. S., Reis, D., Barros, M. V. G., Villalva, M. G. & T. A. D. S., & Theoretical and behavioral analysis of power optimizers for grid-connected photovoltaic systems. *Energy Rep.* **8**, 10154–10167. 10.1016/j.egyr.2022.07.154 (2022).

[24] Karimi-Davijani, H. & Ojo, O. Dynamic operation and control of a multi-DG unit standalone Microgrid, ISGT Anaheim, CA, USA (2011). 10.1109/ISGT.2011.5759177.

[25] Liu, F., Duan, S., Liu, F., Liu, B. & Kang, Y. A variable step size INC MPPT method for PV systems. *IEEE Trans. Industr. Electron.* **55** (7), 2622–2628. 10.1109/TIE.2008.920550 (2008).

[26] Elmahallawy, M., Elfouly, T., Alouani, A. & Massoud, A. M. A comprehensive review of lithium-ion batteries modeling, and state of health and remaining useful lifetime prediction. *IEEE Access* **10**, 119040–119070. 10.1109/ACCESS.2022.3221137 (2022).

[27] Koseoglou, M. et al. A lithium-ion battery equivalent circuit model based on a hybrid parametrization approach. *J. Energy Storage*. **73**, 109051. 10.1016/j.est.2023.109051 (2023).

[28] Castro, M. T. et al. Multiphysics modeling of lithium-ion, lead-acid, and vanadium redox flow batteries. *J. Energy Storage*. **42**, 102982. 10.1016/j.est.2021.102982 (2021).

[29] De Brabandere, K. et al. A voltage and frequency droop control method for parallel inverters. *IEEE Trans. Power Electron.* **22** (4), 1107–1115. 10.1109/TPEL.2007.900456 (2007).

[30] Guerrero, J. M., Vasquez, J. C., Matas, J., de Vicuna, L. G. & Castilla, M. Hierarchical control of droop-controlled AC and DC microgrids—a general approach toward standardization. *IEEE Trans. Ind. Electron.* **58**, 158–172. 10.1109/TIE.2010.2066534 (2011).

